# Adequate vs. complete vs. full house surgery: What are we really talking about?

**DOI:** 10.1016/j.bjorl.2025.101687

**Published:** 2025-07-11

**Authors:** Miguel Soares Tepedino, Renato Roithmann, Fabiana Cardoso Pereira Valera, Fabrizio Ricci Romano, Eduardo Macoto Kosugi, Otávio Bejzman Piltcher, Leonardo Lopes Balsalobre Filho, Edwin Tamashiro, Wilma Terezinha Anselmo-Lima

**Affiliations:** a Universidade do Estado do Rio de Janeiro, Faculdade de Ciências Médicas, Rio de Janeiro, RJ, Brazil; b Universidade Luterana do Brasil, Canoas, RS, Brazil; c Universidade de São Paulo (USP), Faculdade de Medicina de Ribeirão Preto (FMRP), Ribeirão Preto, SP, Brazil; d Universidade de São Paulo (FMUSP), Faculdade de Medicina (FM), São Paulo, SP, Brazil; e Universidade Federal de São Paulo (UNIFESP), Escola Paulista de Medicina (EPM), São Paulo, SP, Brazil; f Faculdade de Medicina da Universidade Federal do Rio Grande do Sul (FAMED-UFRGS), Porto Alegre, RS, Brazil

## Introduction

Endoscopic sinus surgery is an essential step in the treatment of chronic rhinosinusitis with nasal polyps (CRSwNP), especially in the type 2 (T2) endotype. Even in the era of personalized medicine and biological therapies, surgery continues to play a central role in reducing the inflammatory burden, restoring ventilation, sinus drainage and most importantly, optimizing the topical delivery of corticosteroids.

In the Brazilian context, this importance is particularly noteworthy: the indication for biologics depends on the prior performance of adequate surgery, according to ANS guidelines and institutional protocols, such as the Brazilian guideline. However, in practice, there is significant variability in the extent and quality of surgeries performed, raising a critical question: when can we truly consider a surgery to be adequate, complete, or even “full house”?

This editorial proposes a reflection on these concepts, which often remain subjective and inconsistent. It emphasizes the importance of establishing objective anatomical criteria to better evaluate surgical outcomes and guide treatment decisions in managing CRSwNP T2.^1^

An anatomical perspective beyond subjectivity: “Adequate,” “Complete,” and “Full House”

Although the terms 'adequate surgery,' 'complete surgery,' and 'full house' are frequently used subjectively and without standardization in the literature, it is both possible and necessary to seek objective criteria to assess the surgery performed.^2^ In this context, we propose that the term 'complete surgery' be reserved for situations in which there was wide opening within the anatomical boundaries of each paranasal sinus approached, allowing effective ventilation and therapeutic access. That is:

• Ethmoid: complete removal of ethmoidal cells, with exposure of the skull base and the medial orbital wall.
• Maxillary: wide antrostomy starting from the natural ostium, respecting anatomical boundaries (lacrimal bone, lamina papyracea, inferior turbinate, posterior wall of the maxillary sinus).
• Sphenoid: wide bilateral sphenoidotomy with full communication with the nasal cavity.
• Frontal: frontal opening proportional to anatomy and disease (ideally Draf IIa and, when specifically indicated, Draf IIb or III).

Thus, even if the term “complete” maintains some subjectivity, its use should be anchored in clear and reproducible anatomical criteria, avoiding the misclassification of insufficient procedures as complete surgeries.

## The risk of incomplete surgery in CRSwNP T2

Several recent studies show that residual ethmoidal cells, narrow antrostomies, frontal septations, partial sphenoidotomies are associated with a higher risk of early recurrence, persistence of active T2 inflammation, early need for revision surgery and failure of topical corticosteroid delivery, including via nasal irrigation.^3^, ^4^, ^5^ The literature reinforces that it is not about performing indiscriminately aggressive surgeries, but rather surgeries that are adequate to the disease’s pathophysiology.

In practice, there is a growing trend toward performing incomplete surgeries that do not respect the basic principles of proper postoperative anatomy. This creates a scenario in which patients are labeled as “surgical failures” and rapidly referred for biological therapies, when in fact they have not undergone a complete and effective surgical procedure.

Recent publications demonstrate that a complete surgery, well performed and aligned with the anatomical criteria discussed in this editorial, can control the disease in a considerable percentage of patients, substantially reducing the need for early escalation to high-cost therapies. Studies indicate that after complete surgery associated with optimized topical corticosteroid irrigation, 50%–70% of patients with CRSwNP T2 achieve adequate disease control.^4^ Conversely, incomplete surgeries show recurrence rates above 50% within 12–24 months, with a consequent significant increase in the need for early escalation to biological therapies.^3^^,^^5^

Therefore, it is imperative that the patient undergoes complete, functional surgery meeting appropriate anatomical criteria before being considered refractory and indicated for biological therapy. Beyond being an ethical responsibility to the patient, this approach directly impacts health resource management and long-term clinical outcomes.

## Conclusion

The goal is not to pursue surgical radicality but to ensure adequate postoperative anatomy for the control of CRSwNP T2. Incomplete surgeries, with obstructed or poorly ventilated sinuses, limit the effectiveness of topical and systemic therapies, compromise prognosis, and burden health systems with avoidable reoperations and therapies. Advancing toward the standardization of anatomical criteria, as discussed in this editorial, is an important step in promoting better care and more consistent outcomes for our patients.

## Declaration of competing interest

The authors declare no conflicts of interest.
